# Emergency Medicine Physician Assistant (EMPA) Postgraduate Training Programs: Program Characteristics and Training Curricula

**DOI:** 10.5811/westjem.2018.6.37892

**Published:** 2018-07-26

**Authors:** Chadd K. Kraus, Terry E. Carlisle, Devin M. Carney

**Affiliations:** * Geisinger Health System, Department of Emergency Medicine, Danville, Pennsylvania; † University of Missouri-Columbia, Department of Emergency Medicine, Columbia, Missouri; ‡ University of Missouri-Columbia, Columbia, Missouri

## Abstract

**Introduction:**

A growing number of formal postgraduate training programs have been established to provide emergency medicine physician assistants (EMPA) with the unique skills and knowledge to work in the emergency department (ED). The objective of this study was to provide an overview of the current state of EMPA postgraduate training and to describe program characteristics and curriculum components.

**Methods:**

We conducted a cross-sectional study of EMPA postgraduate training programs using data from websites and contacting individual programs to provide program characteristics and curriculum components. Variables collected included length of program, curriculum (e.g., clinical rotations, didactic experience, and research opportunities), size of program/number of trainees, affiliation with emergency medicine (EM) residency, geographic location, and salary.

**Results:**

We identified 29 EMPA postgraduate training programs in 17 states, with at least one additional program in development. The mean length of EMPA training programs is 15 months (range 12–24 months). The most common non-ED/elective rotations are orthopedics, ultrasound, anesthesiology, and trauma. The mean number of trainees per class is 3.46 (median 3, range 1–16 trainees); 27 of 29 (93%) programs were in institutions that also had an EM residency program. The mean annual salary is $58,566 (range $43,000–90,000).

**Conclusion:**

EMPA postgraduate training programs have common characteristics and curriculum components despite a lack of a specialty-specific accrediting organization or certifying examination. The overall growth and current number of these programs merits further research focusing on whether standardized curricula, formal recognition, and accreditation should be developed.

## INTRODUCTION

Physician assistants (PAs) have been integrated into clinical practice in emergency departments (ED) since the early 1970s,^1^ predating the recognition of emergency medicine (EM) as a specialty by the American Board of Medical Specialties. According to the National Commission on the Certification of Physician Assistants (NCCPA), there are over 12,000 certified PAs working in EM, representing 13% of all certified PAs.^2^

There are currently more than 225 Accreditation Council for Graduate Medical Education-accredited EM residencies,^3^ yet workforce projections suggest that fully staffing emergency departments (ED) with residency-trained, board-certified emergency physicians (EP) will continue to be a challenge for the foreseeable future.^4^,^5^ The workforce mismatch is particularly pronounced in rural settings. For example, in Iowa less than 12% of all EDs are staffed exclusively with EPs.^6^ Many EDs, including pediatric and academic EDs, use PAs to augment the EP workforce.^7^–^11^ Emergency medicine physician assistants (EMPAs) play an increasingly important part of the EM patient care team for a variety of presentations,^12^ and can positively impact department productivity^13^ and throughput.^14^

Unlike EPs, EMPAs are not required to complete formal postgraduate training programs in EM, and currently there are no EM-specific standards, competencies, or continuing education requirements for EMPAs. Almost 80% of EMPAs cite “on-the-job training” as the method by which they receive EM training.^15^ Increasing EM training and educational opportunities is beneficial to the development of the EMPA workforce and has been cited as critical to the future of EM.^16^

EMPA postgraduate training programs offer an opportunity to formalize training in EM for PAs and to provide a foundation for lifelong learning and practice improvement. Completion of specialty-specific postgraduate training by PAs has been identified as an alternative to on-the-job training, and might enhance competitiveness in the job market and decrease onboarding time for newly hired PAs.^17^ However, there is variability in the definition of the training and in the structure and standardization of these clinical training experiences.^18^

Postgraduate training programs for PAs have existed in multiple specialties since the early 1970s, and since at least the 1980s for EMPAs.^19^,^20^ An early, prototype EMPA program began in the late 1980s at the University of Southern California/Los Angeles County Hospital. This program offered the opportunity for PAs to develop specialized training in the knowledge and skills that are unique to the practice of EM, and helped lead to the formation of the Society for Emergency Medicine Physician Assistants (SEMPA).^21^ Over the past three decades, EMPA training programs developed and evolved along with the specialty of EM. The objective of this study was to provide an overview of the current state of EMPA postgraduate training and to describe program characteristics and curriculum components.

## METHODS

We performed a cross-sectional study to identify EMPA postgraduate training programs and to describe their characteristics and curriculum components. The study received exempt status approval from the University of Missouri-Columbia School of Medicine Institutional Review Board. Between October 2016 and January 2017, we collected data by searching individual program websites, the SEMPA website, and the Association of Postgraduate PA Programs website. Where incomplete, and to verify information, these public data were supplemented by contacting EMPA programs by telephone and/or email for additional information.

All three authors extracted data using a data form based on an initial review of common program features using a standardized electronic data form (see online appendix). Variables collected included the following: length of program; curriculum (e.g., clinical rotations, didactic experience, and research opportunities); size of program/number of trainees; affiliation with EM residency; geographic location; and salary. We performed descriptive statistical analyses using Excel (Microsoft Corporation, Redmond, Washington).

## RESULTS

We identified a total of 29 EMPA postgraduate training programs, with at least one additional program in development. EMPA programs are found in 17 states (Figure). The mean length of EMPA training programs is 15 months (range 12–24 months). In addition to ED experiences, most programs have a curriculum that includes non-ED (i.e., off-service) required or elective rotations (Table). The most common of these were orthopedics (19/29, 66%), ultrasound (19/29, 66%), anesthesiology (18/29, 62%), and trauma (15/29, 52%). A dedicated pediatric ED experience was offered by 25/29 (86%) of programs. Twenty-two (76%) programs offer or require scholarly activity or research projects. Simulation experience is included in 18/29 (62%) of programs. All programs had didactic conferences that EMPA trainees attended with resident physicians, and most programs (21/29, 72%) had journal club. Additionally, 26/29 (90%) of programs award certificates/diplomas, and 27 of 29 (93%) programs are in departments or divisions of EM or institutions that also had an EM residency program.

The mean annual salary is $58,566 (range $43,000–90,000). The mean number of trainees per class is 3.46 (median 3, range 1–16 trainees). All programs require a formal application process that includes letters of recommendation and an interview. Programs uniformly require certification by the National Commission on Certification of Physician Assistants (NCCPA) prior to beginning the postgraduate EMPA training.

## DISCUSSION

EMPAs are now an important part of the EM workforce and will continue to be in the coming decades. Postgraduate EMPA training programs focused on EM knowledge, skills, and abilities can play a significant role in creating this workforce. EM is a unique practice environment for PAs, featuring a vast knowledge base and procedural competency in a high-risk environment.^22^ EPs gain experience, expertise, and clinical mastery through rigorous residency training and board certification and re-certification examinations and lifelong learning through maintenance of certification activities. A majority of EMPAs develop their EM knowledge, skills, and abilities through on-the-job experiences.

There is an opportunity for the development of standardized curricula, training programs, and a certification process for EMPAs that could be like EP training, with a focus on the unique and collaborative practice needs of EMPAs. This formalized training in EM knowledge and skills would prepare PAs to work in a variety of ED settings. Postgraduate EMPA training programs, when partnered with an EM residency training program, might enhance the understanding of all members of the EM team about the key role of EMPAs in providing emergency care. Further standardization of basic curricula, with the ability to tailor specific program and institutional needs and resources, could accelerate the growth of EMPA training programs by encouraging existing EM residencies to develop co-existing EMPA postgraduate training programs. In 2007, there were five postgraduate EMPA training programs.^23^

Our study identified 29 programs, an increase of 24 programs in a decade. The rapid growth of these programs suggests a need for specialized EM training and is analogous to the increased number of EM residency programs over the past decade. When EM residencies were first developed and board certification adopted as the standard for EPs, the American College of Emergency Physicians (ACEP) played a significant role. Similarly, SEMPA has been at the forefront of education and advocacy for EMPAs, and as a close partner of other EM organizations, including ACEP, could be a catalyst for formalizing EMPA education and certification.

Our study shows curricular similarities among EMPA postgraduate training programs. The curricular similarities that already exist provide a foundation for standardizing EMPA postgraduate training and for the future development of board certification or another similar recognition for EMPAs who have completed these training programs. This formal recognition, whether via board certification or another mechanism would underscore the unique role and commitment of EMPAs to patient care in the ED. Accreditation and certification processes would need to be developed and implemented. One existing example is the availability of voluntary accreditation through the Accreditation Review Commission on the Education for the Physician Assistant (ARC-PA).^24^

Finally, the geographic distribution of EMPA postgraduate programs and EM residency programs are similar, consistent with the affiliation of most EMPA programs in departments and/or institutions with existing EM residencies. EMPA programs are likely found in these settings because of the educational infrastructure and ED volume and acuity that provide the necessary training environment for both EMPA and EM residency training programs. The co-existence of EMPA and emergency physician training programs in the same institution offers an opportunity for interdisciplinary learning and is critical to developing a workforce of EMPAs and EPs who will work collaboratively to provide high quality, patient-centered emergency care.

Our study does not evaluate potential barriers to EMPA training programs or explore outcome measures related to the training. For example, we did not evaluate whether EMPAs who completed postgraduate training provided higher quality, higher value emergency care, or are more desirable as job applicants compared to those who did not have these experiences. We also did not evaluate the potential funding barriers to EMPA programs, especially at a time when government and institutional funding for graduate medical education and postgraduate training is limited, and entering a training program means deferred income for a PA. Future research should explore the cost-effectiveness of EMPA postgraduate training programs in recruiting, training, and retaining EMPAs. Additionally, future investigation into outcomes associated with EMPA postgraduate training is merited.[Table t1-wjem-19-803]

## LIMITATIONS

Our methodology relied primarily on publicly available data and on responses by individual programs to requests for information. It is possible that the data about existing programs are incomplete or missing. Not all postgraduate EMPA programs have websites or other easily accessible information. Because there is no single accreditation body or database for EMPA programs, there might be programs for which data were not included in our study. Our results might underestimate the number of training programs and under-report some of the characteristics of the programs identified. However, the data that are readily available and included in our study suggest similarities among EMPA postgraduate programs from which broad inferences might be drawn about the overall state of EMPA training.

## CONCLUSION

Our results provide a foundation for further investigation of curricular best practices and standardized curricula for EMPA postgraduate training. EMPA postgraduate training programs have common characteristics and curriculum components despite a lack of a specialty-specific accrediting organization or certifying examination. The overall growth and current number of these programs merits further research focusing on whether standardized curricula, formal recognition, and accreditation should be developed specifically for EMPA training programs. Additionally, best practices for program components and educational methods should be evaluated and disseminated among existing programs. EMPA postgraduate training programs can provide an important foundation for expanding the PA workforce in emergency medicine.

## Supplementary Information



## Figures and Tables

**Figure:**
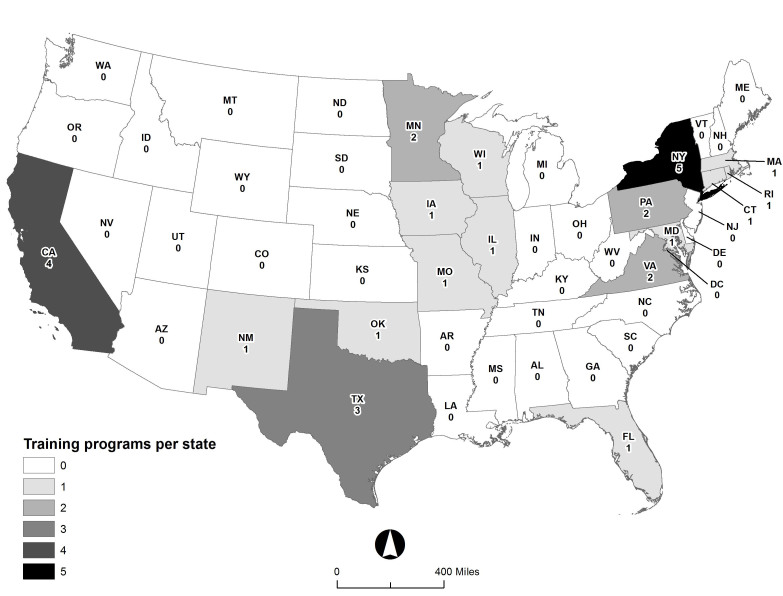
Geographic distribution of emergency medicine physician assistant (EMPA) postgraduate programs.

**Table t1-wjem-19-803:** Clinical experiences (elective or required outside of adult emergency department).

	Number of programs	% of all programs (N = 29)
Pediatrics		
Emergency medicine	25	86
General	3	10
Pediatric intensive care unit	1	3
Orthopedics	19	66
Ultrasound	19	66
Anesthesia	18	62
Medical intensive/critical care unit	17	59
Trauma	15	52
Radiology	13	45
Cardiology (including cardiac intensive care)	12	41
Ophthalmology	11	38
Emergency medical services (ground and/or aeromedical)	10	34
Toxicology	10	34
Obstetrics-gynecology	8	28
General internal medicine	4	14
Neurology	3	10
Other surgical		
Surgical intensive care unit	8	28
Burn	5	17
General surgery	4	14
Oral/maxillofacial	2	7
Wound care	1	3
Miscellaneous		
Wilderness medicine	2	7
Rural medicine	2	7
Disaster medicine	2	7

## References

[1] Podgorny G (1980). Emergency physician assistants: an important adjunct. Ann Emerg Med.

[2] National Commission on Certification of Physician Assistants (NCCPA) (2017). 2016 Statistical Profile of Certified Physician Assistants by Specialty: An Annual Report of the National Commission on Certification of Physician Assistants.

[3] American Board of Emergency Medicine (ABEM) Accredited Training Programs.

[4] Reiter M, Wen LS, Allen BW (2016). The emergency medicine workforce: profile and projections. J Emerg Med.

[5] Ginde AA, Sullivan AF, Camargo CA (2009). National study of the emergency physician workforce, 2008. Ann Emerg Med.

[6] Groth H, House H, Overton R (2013). Board-certified emergency physicians comprise a minority of the emergency department workforce in Iowa. West J Emerg Med.

[7] Menchine MD, Wiechmann W, Rudkin S (2009). Trends in midlevel provider utilization in emergency departments from 1997 to 2006. Acad Emerg Med.

[8] Doan Q, Sabhaney V, Kissoon N (2012). The role of physician assistants in a pediatric emergency department: a center review and survey. Pediatr Emerg Care.

[9] Doan Q, Sabhaney V, Kissoon N (2011). A systematic review: The role and impact of the physician assistant in the emergency department. Emerg Med Australas.

[10] Wiler JL, Rooks SP, Ginde AA (2011). Update on midlevel provider utilization in US emergency departments: 2006 to 2009. Acad Emerg Med.

[11] Hooker RS, Klocko DJ, Larkin GL (2011). Physician assistants in emergency medicine: the impact of their role. Acad Emerg Med.

[12] Ginde AA, Espinola JA, Sullivan AF (2010). Use of midlevel providers in US EDs, 1993–2005:implications for the workforce. Am J Emerg Med.

[13] Brook C, Chomut A, Jeanmonod RK (2012). Physician assistants’ contribution to emergency department productivity. West J Emerg Med.

[14] Nestler DM, Fratzke AR, Church CJ (2012). Effect of a physician assistant as triage liaison provider on patient throughput in an academic emergency department. Acad Emerg Med.

[15] Arbet S, Mauldin S (2009). Society of Emergency Medicine Physician Assistants (SEMPA). Report on Emergency Medicine Physician Assistants.

[16] Schneider SM, Gardner AF, Weiss LD (2010). The future of emergency medicine. Ann Emerg Med.

[17] Will KK, Williams J (2016). Perceived efficacy and utility of postgraduate physician assistant programs. JAAPA.

[18] Hussaini SS, Bushardt RL, Gonsalves WC (2016). Accreditation and implications of clinical postgraduate PA training programs. JAAPA.

[19] Herrera J, Gendron BP, Rice MM (1994). Military emergency medicine physician assistants. Mil Med.

[20] Sturmann KM, Ehrenberg K, Salzberg MR (1990). Physician assistants in emergency medicine. Ann Emerg Med.

[21] Kraus CK (2017). EM Physician Assistant Terry Carlisle Recall Long Career, Discusses Future of Specialty. ACEPNow.

[22] Klauer K (2013). Innovative staffing in emergency departments: the role of midlevel providers. CJEM.

[23] Jones PE (2007). Physician Assistant Education in the United States. Acad Med.

[24] (2018). Accreditation Review Commission on the Education for the Physician Assistant.

